# Are realistic details important for learning with visualizations or can depth cues provide sufficient guidance?

**DOI:** 10.1007/s10339-024-01183-3

**Published:** 2024-03-21

**Authors:** Alexander Skulmowski

**Affiliations:** Digital Education, Institute for Informatics and Digital Education, Karlsruhe University of Education, Bismarckstr. 10, 76133 Karlsruhe, Germany

**Keywords:** Realism, Learning, Visualization, Cognitive load, 3D

## Abstract

The optimal choice of the level of realism in instructional visualizations is a difficult task. Previous studies suggest that realism can overwhelm learners, but a growing body of research demonstrates that realistic details can enhance learning. In the first experiment (*n* = 107), it was assessed whether learning using realistic visualizations can be distracting and therefore particularly benefits from pre-training. Participants learned the anatomy of the parotid gland using labeled visualizations. While pre-training did not have an effect, a more realistic visualization enhanced learning compared to a schematic visualization. In the second experiment (*n* = 132), a schematic diagram was compared to a more realistic style featuring basic depth cues, and a highly realistic visualization containing a detailed surface. Regarding retention performance, no significant differences were found. However, an interesting pattern regarding subjective cognitive load ratings emerged: the schematic version received the highest cognitive load ratings, while the version featuring simplified shading was rated as least demanding. The version containing simplified depth cues also elicited lower cognitive load ratings than the detailed visualization. The two experiments demonstrate that fears concerning a detrimental effect of realistic details should not be over-generalized. While schematic visualizations may be easier to visually process in some cases, extracting depth information from contour drawings adds cognitive demands to a learning task. Thus, it is advisable that computer-generated visualizations contain at least simplified forms of shading, while the addition of details does not appear to have a strong positive effect.

**Supplementary Information:**

The online version contains supplementary material available at 10.1007/s10339-024-01183-3.

## Introduction

In the last several years, there has been an increasing interest in the effects of realism in visualizations on learning. While this topic has been investigated for decades (e.g., Dwyer 1967, 1969; Scheiter et al. 2009), older studies needed to rely on comparisons of analogue media, such as drawings and various types of photographs. A plethora of studies investigated the effects of realistic details on learning, for instance by varying the level of realism in instructional visualizations (e.g., Dwyer 1967, 1969). Other studies dealt with the interactions between contextual factors, such as learners’ prior knowledge (Dwyer 1975). Due to the growing usage of digital learning, ranging from websites featuring three-dimensional (3D) computer-generated visualizations to virtual reality, learners and educators need to know which presentation mode(s) will help them reach their learning objectives. As a result, the recent years saw a revival of this research area.

Research on learning with realistic visualizations is encumbered by a number of obstacles that have impeded researchers in coming to broader conclusions and recommendations. A theoretical problem persists in the definition of realism in computer-generated visualizations. While some studies focus on comparisons between the extreme opposites of “schematic” (or “abstract”) visualizations and “realistic” (or “detailed”) visualizations (e.g., Menendez et al. 2020, 2022; Scheiter et al. 2009; Skulmowski and Rey 2020), there have been various attempts at defining discrete realism levels that often range from the abstraction level of contour drawings to photorealistic visualizations (e.g., Dwyer 1967; Höffler 2010). Although such systems provide some guidance for the categorization and comparison of learning materials used in different studies, it may still be difficult to reliably label different studies as belonging to a certain level. This problem is pervasive in instructional realism research and has been discussed as a major issue before (Skulmowski and Rey 2018; Skulmowski et al. 2022). After all, if there is no agreement on what constitutes the different levels of realism, it is impossible to agree on whether there can be an optimal level of realism.

**Fig. 1:**
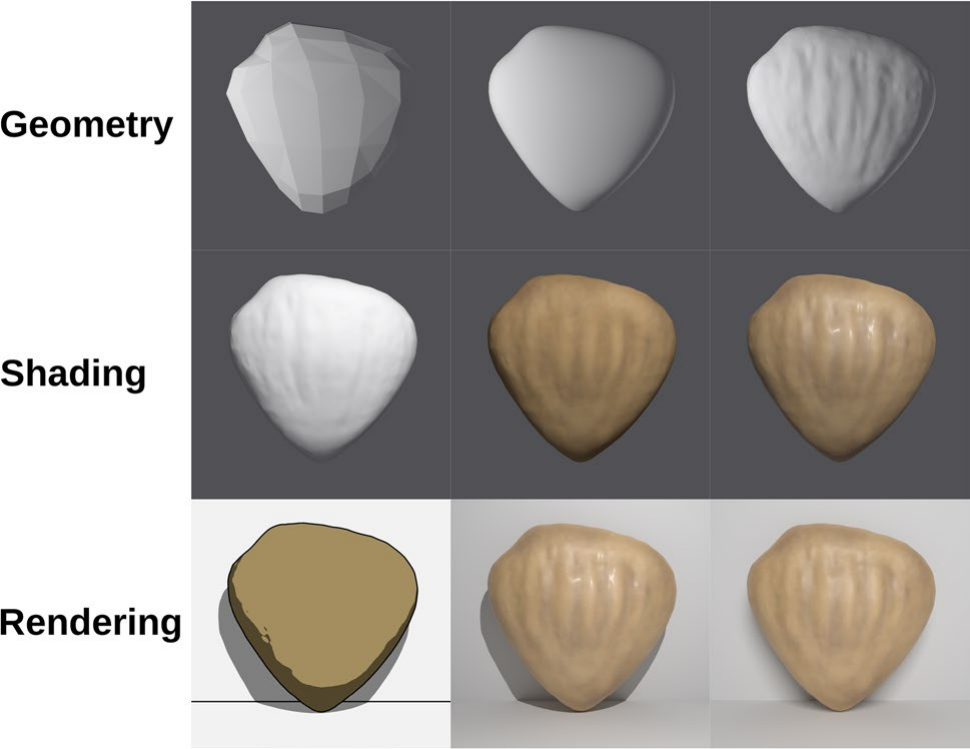
The geometry, shading, and rendering (GSR) model developed by Skulmowski et al. (2022). The visualizations used for the experiments in this paper vary the level of realism on the three dimensions involved in the actual creation of computer-generated visualizations. By creating a more or less detailed geometry, the number of elements that learners need to attend to is determined, but also the styling and fidelity of the virtual model to a real counterpart. Using shaders, virtual materials that utilize color maps and other surface qualities can be developed. Shading can also be used to add further details to a model. The rendering options in current visualization software offer the choice between simplification and physical realism (and levels in-between). Furthermore, the placement of virtual lights can greatly affect the look of a rendering. From “Is a Preference for Realism Really Naive After All? A Cognitive Model of Learning with Realistic Visualizations,” by A. Skulmowski, S. Nebel, M. Remmele, and G. D. Rey, 2022, *Educational Psychology Review*, *34*, p. 656 (https://doi.org/10.1007/s10648-021-09638-1). © 2021 Skulmowski, Nebel, Remmele, and Rey (licensed under the Creative Commons Attribution License, http://creativecommons.org/licenses/by/4.0)

Earlier research often used the idea of a “realism continuum” to distinguish between several levels of realism (e.g., Dwyer 1967). However, more specialized methods of categorization have been presented for the realm of computer-generated visualizations. Slater et al. (2009) defined realism using the two components *geometric realism* and *illumination realism*. The former component is defined as the result of the virtual model depicted having a geometry that captures the real model as accurately as possible, while the latter component is realized by using physically correct lighting calculations to let the geometry appear as it does in real life. A more detailed system was presented by Skulmowski et al. (2022) with the geometry, shading, and rendering (GSR) model (see Fig. 1). The model considers the three major steps in creating a computer-generated visualization: starting with the level of detail of the geometry, followed by the various options concerning the appearance of the materials applied to the models in the shading stage, and concluding with the lighting and rendering stage that is used to determine the look of the rendering (ranging from a drawing-like schematic output to photorealistic renderings).

Even when studies are carried out by the same researcher(s) using the same learning materials, such as in Francis Dwyer’s case, who conducted a number of studies on learning heart anatomy and physiology (see Dwyer 1976, for an overview); the effectiveness of realism can vary substantially between studies (for a meta-analysis and discussion, see Reinwein and Huberdeau 1997). As noted by Dwyer (1976), factors such as the learning time, learners’ prior knowledge, and the learning objectives can affect the usefulness of realism. In sum, realism can be considered to be difficult to define and categorize, and even with clearly defined realism levels, the effects of realism do not appear to be consistent.

### Are realistic details a form of distraction?

With the growing number of studies in which realism did not have a significant effect or even resulted in negative effects on performance, some authors characterized the belief that realism can be helpful as naive (Smallman and St. John, 2005). Despite such drastic conclusions, a new wave of realism research focused on computer-generated instructional visualizations has contributed to a more balanced view (e.g., Huk 2006; Huk et al. 2010; Menendez et al. 2022; Moreno et al. 2011; Skulmowski 2022a, 2022b; Skulmowski and Rey 2020, 2021). These studies highlight that visual realism can be particularly helpful for learners with high spatial abilities (Huk 2006), for learners of specific ages (Menendez et al. 2022), and for realistic tests (Skulmowski and Rey 2021).

Turning to relevant reviews, it becomes apparent that realism (or a large amount of detail, often called *perceptual richness*) resulted in mixed results, but also appears to help learners accomplish specific goals (Cromley and Chen 2023; Fyfe et al. 2014; Skulmowski et al. 2022). The overall conclusion that can be gained from these reviews is that more abstract cognitive processes (such as comprehension or drawing inferences, e.g., Butcher 2006; Kaminski and Sloutsky 2013; Kaminski et al. 2008, 2013) do not benefit from realistic details (or are even hindered by them), while tasks centered around learning concrete and visual information can gain from realism (e.g., Skulmowski 2022a, 2022b). This pattern of results provides strong evidence for the claim that realism can be beneficial if utilized appropriately. However, the complexity of this pattern also highlights that still not enough is known about the effects of realism on learning to provide straightforward guidelines.

A recurring criticism toward the use of realistic instructional visualizations is that details may be unnecessary and overwhelming (e.g., Scheiter et al. 2009; for an overview, see Skulmowski et al. 2022). According to Skulmowski et al. (2022), learners using realistic visualizations may be facing the challenge of dealing with a certain level of perceptual load stemming from details, resulting in a higher cognitive load during learning. As learners need to distinguish which details are relevant and which are not, this cognitive load has been characterized as a form of *extraneous cognitive load* as defined by Sweller et al. (1998, 2019) in previous research (e.g., Scheiter et al. 2009). In the framework of cognitive load theory, extraneous cognitive load is a theoretical container for all cognitive demands that are unrelated and distracting in a learning task (Sweller et al. 1998). As acknowledged in this theory, learners only have a working memory with a very limited capacity at their disposal. Extraneous cognitive load prevents learners from investing their working memory capacity in the acquisition of relevant information, the latter being called *intrinsic cognitive load* (Sweller et al. 1998). While the boundaries between working memory and sensory memory are hard to draw (e.g., Guo et al. 2021; for an overview, see Shevlin 2020), research on multimedia learning typically assumes the distinct memory stores of sensory memory, working memory, and long-term memory (e.g., Mayer 2014; for an overview, see Schweppe and Rummer 2014). In this view, perceptual load stemming from visually complex realistic details can be thought of as the precursor to cognitive load in the form of detailed visual elements that need to be kept in working memory (Skulmowski et al. 2022). Thus, a high perceptual load stemming from irrelevant realistic details could be assumed to contribute toward extraneous cognitive load. Although it is generally recommended to minimize extraneous cognitive load in order to optimize instruction (e.g., Sweller 2020), research has shown that higher subjective extraneous cognitive load scores do not necessarily go hand in hand with a lower learning performance (e.g., Skulmowski 2022a), making a prediction of the effects of realism even more difficult.

A recent series of studies found mixed evidence for the assumption that realism can act as a distracting influence (Skulmowski 2023a). In both studies of that paper, realism was contrasted with the split-attention effect (i.e., the finding that scattering relevant information leads to worse learning than keeping related information in close proximity, Chandler and Sweller 1991, 1992). The studies were conducted to assess whether realism and split attention reinforce each other in a negative way, which would have suggested that these two design features act on shared processing pathways. In both studies, realism did not exacerbate the negative effects of split attention, but independently had no or a negative effect on learning (in Experiment 1 and 2, respectively). Upon closer inspection of the realistic visualization used in Experiment 2 (Skulmowski 2023a), the negative effect of this particular realistic visualization could be attributed to the numerous shiny details that do not provide sufficient semantic information in return for their perceptual demands (for related discussions, see Skulmowski 2023b; Skulmowski and Xu 2022).

In research on instructional visualizations, detailed realistic renderings and simple line drawings are often considered to be the extreme ends of the realism spectrum (Skulmowski et al. 2022). Hertzmann (2020) recently proposed to reconsider this contrast and instead regard drawings consisting of contour lines as a simplified substitute of reality. In Hertzmann’s (2020) model, generating a line drawing can be thought of as removing all surface details other than object boundaries. Perceiving a line drawing, on the other hand, involves generating inferences about the 3D form of an object (Hertzmann 2020). Following this proposal, contour lines could be considered as a way of presenting a wealth of visual information in a compressed form that can be “unpacked” by the viewer in order to generate a 3D mental representation. According to Hertzmann (2020), this mental ability of constructing a 3D representation from contour lines can be an automatic step performed in the visual system for simple contours or an ability that needs to be trained, depending on the style of the visualization. However, as these steps appear to be relatively cognitively demanding, one might argue that such an “unpacking” process may add additional demands, resulting in a higher cognitive load through simplification.

In sum, several results and theoretical considerations contributed toward a mixed pattern of results regarding the impact of realism on learning. While realism often plays a key role in achieving specific learning objectives, the perceptual demands of a high number of details can be overwhelming and unnecessary for other learning tasks. However, the perceptual demands of inferring a complex 3D shape from a simplified outline (see Hertzmann 2020) may also contribute toward cognitive load. As a result, a closer investigation concerning the main drivers behind perceptual demands in learning with visualizations is necessary. In addition, given the remaining potential for distraction inherent in realistic and detailed instructional visualizations, the question arises whether this danger can be averted by enriching a learning task with a pre-training phase. An overview of the effects of pre-training is given in the following section.

### Realism and prior knowledge in sequential processing

Prior knowledge is an important aspect to consider in the design of a learning task (for overviews, see Brod 2021; Mayer and Fiorella 2021). Mayer et al. (2002) used the pre-training principle in a way that subdivides a complex learning task into two easier ones: based on the assumption that an animation explaining the mechanism behind brakes would be too complex, they were successful with an approach that lets learners explore the components depicted in the animation first, and then presenting the narrated animation. As this animation highlighted the causal relationships between the parts, learners who completed the pre-training had more cognitive resources to focus on these relationships than those who did not (Mayer et al. 2002; Mayer and Moreno 2003).

In the context of realism research, Dwyer (1975) found in a quasi-experimental study that a high level of prior knowledge benefits learners regardless of the level of realism used in the learning task, but that learners with a low and medium level of prior knowledge struggle with more realistic visualizations. Based on the results discussed in this section, the factor of prior knowledge could be used to test the claim that realism is able to induce so much perceptual load as to distract learners from other information. Using the pre-training principle, a typical anatomy learning task in which learners need to memorize what an anatomical structure looks like and how the components are named can be broken down into a sequence of two steps: (1) Learn using a text in which the components are described and named; (2) learn using the complete labeled visualization. If the claim that realism is detrimental due to a distractive influence is true, there should be a particularly strong positive effect on learning with a pre-training intervention if a realistic rather than a schematic visualization is used for the second step of such a learning task. In other words, pre-training could compensate the potential negative effects of realism.

### The present studies

In the first experiment, pre-training is used to assess whether realistic visualizations distract learners by keeping their attention off of the labels. For learners who receive a short text mentioning the names of the different parts and explaining their shape, this type of pre-training should be particularly beneficial if they are learning with the realistic rather than the schematic version of the visualization. Thus, an interaction effect between the factors pre-training (without versus with) and realism (schematic versus realistic) was assumed (H_1a_). Regarding the effect on extraneous cognitive load, an inverse relationship of this interaction effect was hypothesized (H_1b_).

The second experiment investigated whether realistic details are needed for a comprehensive mental representation or whether depth cues—lacking the distractive potential of detailed renderings—are sufficient. If the positive effects of realism stem from depth cues, the variant containing such cues should lead to a significant increase in retention performance compared with the schematic drawing (H_2a_). The realistic version should have an even stronger positive effect on retention than the version containing depth cues (compared to the schematic drawing) if surface detail is indeed relevant for learning (H_2b_). Based on related research (Skulmowski 2022a), it was assumed that the level of subjective extraneous cognitive load rises with more realism, so that depth cues (H_3a_) and realistic details (H_3b_) result in higher cognitive load ratings than the schematic version.

## Experiment 1

### Method

#### Participants and design

As previous effect sizes in realism research using a similar methodology resulted in medium to high effect sizes (*η*p^2^) between 0.09 (Skulmowski 2022b) and 0.14 (Skulmowski and Rey 2021), and a recent study investigating the pre-training principle in virtual reality indicated a similarly large effect size of *d* = 0.62 (Meyer et al. 2019), a conservative estimate of *η*p^2^ = 0.07 was chosen as the basis for the sample size calculation.^1^ Using G*Power (Version 3.1.9.2; Faul et al 2009), a sample size of 107 was calculated for the 2 × 2 design of this study (power = 0.80). The two between-subjects factors investigated in this experiment are realism (schematic versus realistic) and pre-training (without versus with).

Participants needed to fulfill certain criteria in order to participate. They needed to be native German speakers aged between 18 and 30 years who had no or little knowledge concerning the anatomy of the parotid gland. In addition, only the data of participants who confirmed that they were not strongly distracted and that no major technical problem had occurred during the learning task at the end of the study were counted as complete datasets to be used for further analyses (as in the study by Skulmowski and Rey 2020). A total of 130 participants took part in the study, with 22 of them not fulfilling the participation criteria and one participant indicating having been strongly distracted, leaving 107 datasets to be analyzed.

Of the 107 participants whose datasets were complete, 90 were female and 17 were male. All participants in the two studies presented in this article were students enrolled in teacher training courses and participated for partial course credit at a university of education in Germany. Using block randomization, three of the groups were assigned with 27 participants, and only the group receiving the pre-training before learning with a schematic visualization contained 26 participants.

#### Materials

**Fig. 2:**
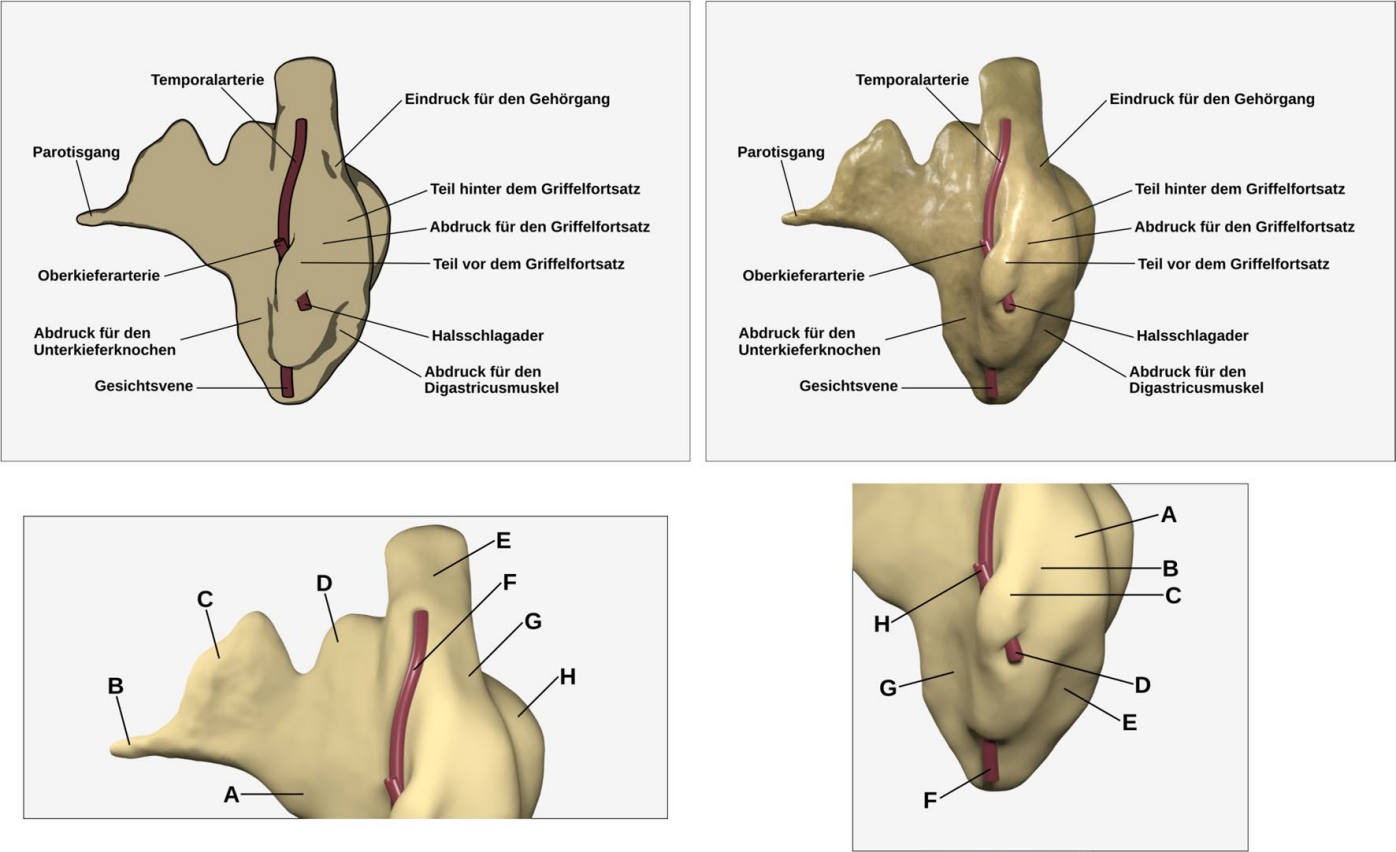
The instructional visualizations used in Experiment 1. The visualizations are revised versions of the images used by Skulmowski and Rey (2021). The top row features the two visualizations used in the learning phase. The left panel shows the schematic version and the right panel displays the realistic version. The bottom row shows the two visualizations used on the two pages of the retention test. Adapted from “Realism as a Retrieval Cue: Evidence for Concreteness-Specific Effects of Realistic, Schematic, and Verbal Components of Visualizations on Learning and Testing,” by A. Skulmowski and G. D. Rey, 2021, *Human Behavior and Emerging Technologies*, *3*, p. 287 (https://doi.org/10.1002/hbe2.209). © 2020 Skulmowski and Rey (licensed under the Creative Commons Attribution License, http://creativecommons.org/licenses/by/4.0)

The experiment used revised versions of the visualizations developed by Skulmowski and Rey (2021). In that study, participants learned the anatomy of the parotid gland either using a realistic or a schematic visualization. Using the source files of the scenes used to generate the renderings, a number of changes were made to the original version to increase the difference between the two visualizations (see Fig. 2, top row). All renderings used for the visualizations in this article were created using Blender (https://www.blender.org). The schematic version presents the parotid gland as a contour drawing filled with solid colors and minimal shading to provide the most important depth cues. The realistic version uses the same base geometry, but features realistic shading involving a color texture, bump mapping, and highlights. For the realistic version, physically correct rendering methods using a lighting setup that provides additional depth cues were employed. Thus, according to the GSR model, there would essentially be no difference in the geometry dimension, but strong contrasts in the shading and rendering dimensions. For the pre-training group, a short text (124 words) was prepared in which the different components shown in the visualization are named, and their location is explained (as in the following translated example, “From this irregularly shaped structure, the parotid duct grows out.”; the full text can be found in the supplementary file).

There are several approaches to designing visual learning tests in realism research. In the design of test visualizations, it needs to be considered whether some types of visualizations lead to biased results (see, e.g., Scheiter et al. 2009, for a discussion). While some studies utilize only schematic visualizations (e.g., Skulmowski 2022b), another approach is to use visualizations that blend schematic and realistic attributes in order to arrive at an “in-between” level that is common to both visualizations (e.g., Skulmowski and Rey 2018). However, it needs to be noted that the original study using the parotid gland visualizations revealed that a benefit of realistic visualizations during learning may only be measurable using an equally realistic test visualization (Skulmowski and Rey 2021). For the present study, an in-between approach was chosen in which the model is rendered realistically (thus, preserving all depth cues), but without a detailed material (see Fig. 2, bottom row). As in the original study, the retention test was divided into two visualizations containing lettered components to which the appropriate names needed to be assigned. Some of these components were not labeled during the learning phase and thus were needed to be assigned the option “NOT LEARNED.” For every correctly labeled component, participants scored one point, with a maximum score of 16 points. Incorrect responses did not result in penalty points. The retention test resulted in a reliability of McDonald’s *ω* = 0.66. The study included the three extraneous cognitive load items from Klepsch et al. (2017) that were presented with the modified wording used by Skulmowski and Rey (2020), therefore asking participants regarding their difficulties while working with the visualization, rather than their rating concerning the entire task. The averaged score of the three items using 7-point Likert scales is used for the analyses in both studies in this paper. In this study, the extraneous cognitive load items had a reliability of *ω* = 0.88. Both studies in this paper used SoSci Survey (Leiner 2021) to collect the data.

#### Procedure

The general procedure is similar to previous studies (e.g., Skulmowski and Rey 2020). The study was conducted in a PC laboratory with ten seats. Participants were required to wear face masks during the study due to COVID-19 regulations in effect at the time. After providing informed consent, participants were asked to provide information regarding the participation criteria (age range between 18 and 30 years, German as a native language, no or little knowledge on the topic). The next page of the survey provided participants with the instructions for the learning phase. For all participants, this page stated that their task would be to learn the names, shapes, and locations of the parts of the parotid that were to be presented on the visualization. They were informed about the time limit of 60 s. The pre-training group received an additional instruction that before this task, they would be presented with a short text they were asked to memorize within 90 s. Thus, they were either presented with the visualization of the parotid or the short pre-training text on the next page. Both pages featured a countdown of the remaining time. After this learning phase, they were directed to a page on which the three extraneous cognitive load question items were presented, followed by a filler task. In this sorting task that lasted 60 s, the 16 German federal states were to be ranked according to their number of universities of applied sciences. On the following two pages, the retention tests were presented. On each page, one of the test visualization was shown at the top and for each lettered component in these images; participants were asked to select the corresponding label from drop-down menus below. They were informed that was no time limit for the tests. Next, participants answered questions regarding their gender and course of study as well as two data quality control questions regarding distractions and technical difficulties. Finally, participants were thanked, and they received further information regarding the study.

### Results

The analyses for Experiment 1 were planned as 2 × 2 analyses of variance (ANOVAs) at a significance level of 0.05. For some variables, the normality of residuals assumption (assessed using Shapiro–Wilk tests) was violated and nonparametric tests using aligned rank transformation (Wobbrock et al. 2011) were run instead.

#### Extraneous load

**Fig. 3:**
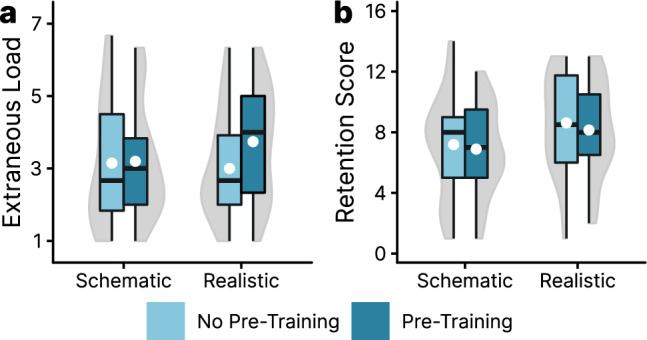
Boxplots with violin elements of the data from Experiment 1. Figure 3a shows the averaged extraneous cognitive load data, and Fig. 3b displays the retention scores. White dots indicate the means of the groups

A nonparametric ANOVA of the extraneous cognitive load data (see Fig. 3a) did not result in significant effects (all *p*s > 0.168). The only tendency of interest was that inducing prior knowledge before learning with the visualization raised the overall extraneous cognitive load on the descriptive level. The hypothesized interaction effect (H_1b_) was not confirmed.

#### Retention

An ANOVA of the retention score data (see Fig. 3b) resulted in a significant benefit of the realistic visualization over the schematic one, *F*(1, 103) = 4.97, *p* = 0.028, *η*p^2^ = 0.05. Prior knowledge and the interaction between the two factors did not result in significant effects (*p*s > 0.528). Thus, H_1a_ did not reach significance and the effect pattern supports the claim that realism does not act as a distractor that needs to be compensated using other instructional means.

## Experiment 2

A second experiment was conducted to assess underlying causes of the strong positive effect of realism on learning found in Experiment 1. The learning materials used in the first experiment compared a schematic version that contains a contour line, a solid halftone fill color, and a solid shadow color. Thus, the schematic version features a limited degree of depth cues through the simplified shading. Still, the realistic version including elaborate shading resulted in better learning scores. The question arises whether the surface details found on the realistic rendering are the cause of this increase in performance (as suggested by Skulmowski and Rey 2021). In order to answer this question, a study comparing a schematic drawing without any depth cues, a simplified rendering with minimal depth cues, and a highly detailed rendering with conspicuous surface detail was conducted.

### Method

#### Participants and design

Based on the result of *η*p^2^ = 0.05 in the first study and the larger effect of *η*p^2^ = 0.14 found by Skulmowski and Rey (2021), a conservative compromise of *η*p^2^ = 0.05 was chosen as the basis for the sample size calculation for the between-subjects design with three factor levels. A sample size of 132 was calculated with G*Power (power = 0.80).

Participants needed to fulfill the same criteria to participate as in Experiment 1 and the same data quality control measures were applied. In total, 141 datasets were generated, with nine participants not fulfilling participation criteria. Of the 132 complete datasets, 111 were obtained from female and 21 from male participants. Through the use of block randomization, 45 participants learned using the schematic drawing, 43 with the rendering featuring depth cues, and 44 participants used the detailed rendering.

#### Materials

**Fig. 4:**
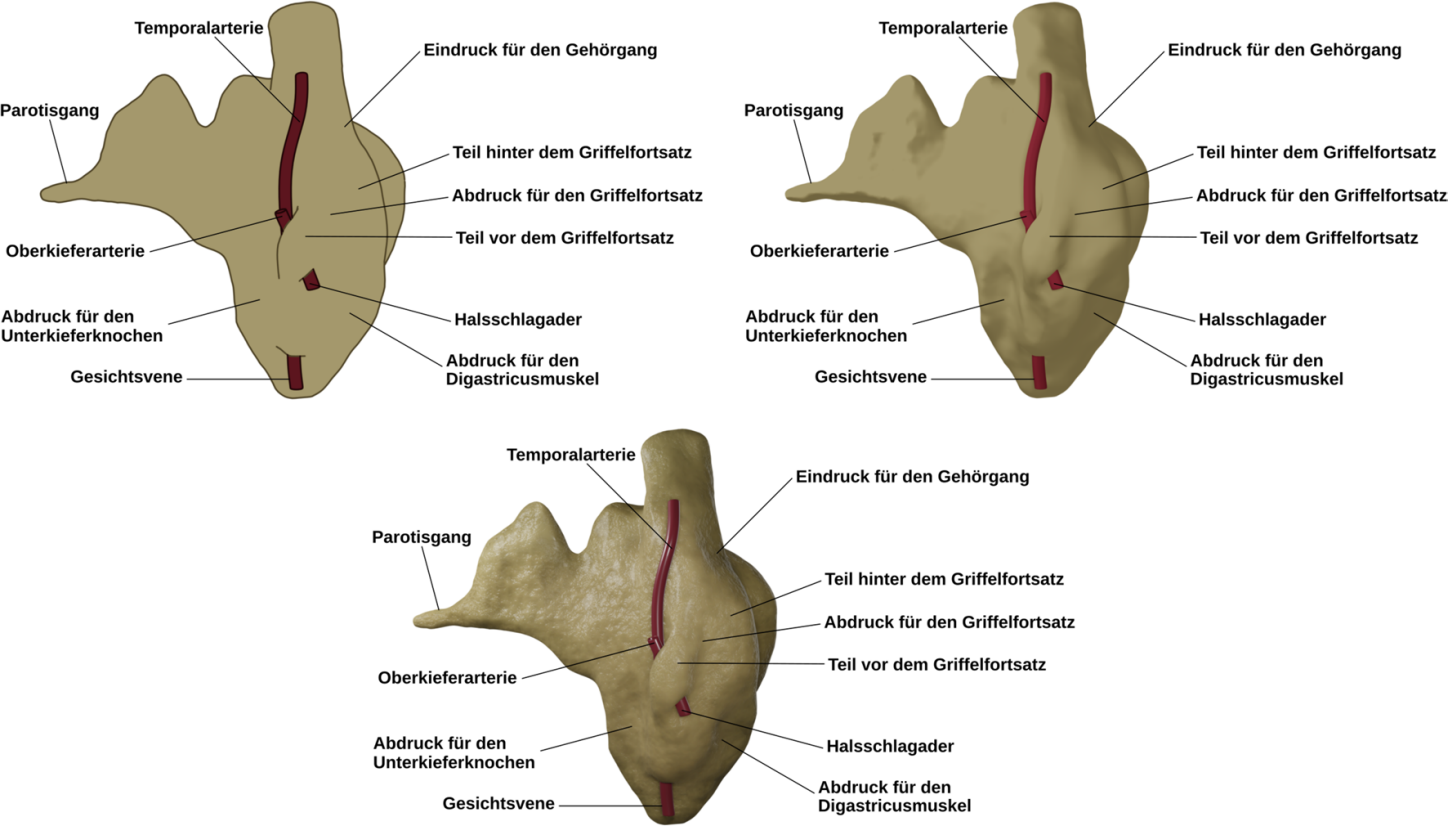
The three instructional visualizations used during the learning phase of Experiment 2. The top row shows the contour drawing (left) and the more realistic rendering featuring depth cues in the form of strongly simplified shadows (implemented through an angle-based gradient shader without highlights). The bottom row presents the detailed rendering, containing details and realistic shading. Adapted from “Realism as a Retrieval Cue: Evidence for Concreteness-Specific Effects of Realistic, Schematic, and Verbal Components of Visualizations on Learning and Testing,” by A. Skulmowski and G. D. Rey, 2021, *Human Behavior and Emerging Technologies*, *3*, p. 287 (https://doi.org/10.1002/hbe2.209). © 2020 Skulmowski and Rey (licensed under the Creative Commons Attribution License, http://creativecommons.org/licenses/by/4.0)

The second experiment also used revised versions of the visualizations developed by Skulmowski and Rey (2021). Three versions featuring strong differences were created (see Fig. 4): (1) a schematic drawing only containing contour lines and solid color fills, (2) a version that shows minimal depth cues through shading, but no highlights, textures, or details, and (3) a realistic rendering with more pronounced detail. The retention test visualizations were largely identical to those used in Experiment 1, but with a slightly different lighting setup. The reliability of the retention test is *ω* = 0.75. The study also used the three extraneous cognitive load items as described for Experiment 1, with a reliability of *ω* = 0.94.

#### Procedure

The general procedure was identical to Experiment 1; however, there was no pre-training stage involved. This study was also conducted in a laboratory.

### Results

The analyses for Experiment 2 were planned as one-factorial ANOVAs with three between-subjects groups. In the case of the extraneous cognitive load data, the normality of residuals assumption was not met and nonparametric tests using aligned rank transformation were used.

#### Extraneous load

**Fig. 5:**
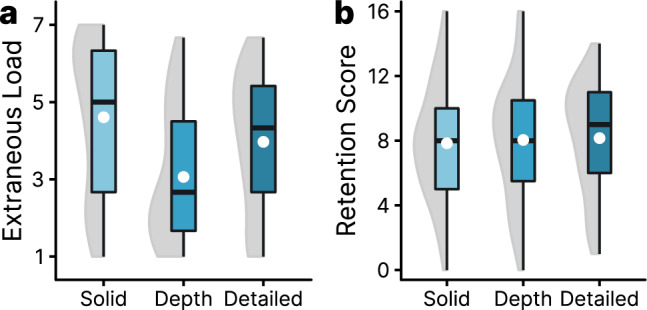
Boxplots with violin elements of the data from Experiment 2. Figure 5a displays the averaged extraneous cognitive load data. Figure 5b shows the retention scores. White dots are used to indicate the means of the groups

A nonparametric ANOVA of the extraneous cognitive load data (see Fig. 5a) resulted in a significant omnibus ANOVA, *F*(2, 129) = 7.74, *p* = 0.001, *η*p^2^ = 0.11. Nonparametric contrasts (Tukey-corrected) demonstrated a significant difference between the extraneous cognitive load ratings for the solid-filled schematic drawing and the version containing depth cues, *t*(129) = 3.93, *p* < 0.001, indicating less cognitive load experienced while learning with depth cues. There was no significant difference between the solid (schematic) and detailed version (*p* = 0.213). The difference between the version with depth cues and the detailed rendering did not result in a significant effect (*p* = 0.070). Considering the high variance of the data (*SD* = 1.95), a robust ANOVA with trimmed means (Wilcox 2017) was performed (like in previous studies on the issue of realism, e.g., Skulmowski and Rey 2020). Using post hoc tests with a trim level of 20%, the difference between the depth cue version and the detailed version reached significance, value = − 1.27, *p* = 0.029.

In sum, the extraneous cognitive load data indicate that providing depth cues leads to a substantially lowered subjective cognitive load compared to a solid-filled visualization without such cues. Compared with the version featuring depth cues, adding realistic details also raises cognitive load. Thus, hypotheses H_3a_ and H_3b_ were not confirmed.

#### Retention

An omnibus ANOVA did not result in a significant effect (*p* = 0.914) and no further tests were run. Consequently, H_2a_ and H_2b_ were not confirmed.

## Discussion

In two laboratory experiments, it was examined how to support learning with computer-generated realistic visualizations. The first experiment revealed that learning with a realistic visualization of the parotid gland results in better retention performance than using a schematic rendering. A pre-training intervention unexpectedly did not have an impact on learning. This result could be interpreted to signify that concerns regarding a distractive effect of realism should not be over-generalized. If realism was as distractive as some authors suggested, a pre-training intervention should have been able to compensate this supposed negative aspect of realism. As this was not the case, we have no conclusive evidence that realism generally distracts learners from other relevant information. While it has been shown that realism can place demands on attention (e.g., Lin et al., 2017; Skulmowski and Rey 2020), these demands may not be so high as to interfere with the learning of other information.

A second experiment was conducted to more closely investigate which specific aspects of realism are beneficial for learners. In this study, a contour drawing was compared to a rendering with simplified depth cues and a highly detailed version. Despite an adequate sample size, there was no significant main effect of realism on retention. On a descriptive level, the schematic version resulted in the lowest retention scores, the version featuring depth cues in a minimally higher mean score, and the detailed version in the highest average score. However, the cognitive load ratings follow a different pattern. The contour drawing received the highest cognitive load scores, followed by the detailed version. The rendering containing simplified depth cues received significantly lower cognitive load ratings than the two remaining versions.

The studies provide evidence against the assumption that realistic details invariably overwhelm learners. Extending previous findings that realistic details are beneficial for tests featuring equally detailed visualizations (Skulmowski and Rey 2021), the first experiment showed that realism raises retention performance overall. Even when these details were amplified in the second study, they did not significantly affect learning. However, the detailed visualization elicited significantly higher cognitive load ratings than the visualizations featuring simpler depth cues, following previously found patterns demonstrating that realistic details increase cognitive load (e.g., Skulmowski 2022a).

The studies provide additional evidence that (certain aspects of) realism can foster learning in specific tasks (see Skulmowski et al. 2022). Similar to other studies investigating how learners process concrete shapes rather than abstract knowledge (e.g., Skulmowski 2022a, 2022b), realism was beneficial compared to a schematic diagram in Experiment 1. Contrary to other studies (e.g., Skulmowski 2022a; Skulmowski and Rey 2020), the perceptual richness induced by realism did not result in a higher subjective cognitive load. However, Experiment 2 revealed additional insights into the factors affecting perceived cognitive load. While a diagram filled with a solid color elicited the highest cognitive load ratings, the lowest cognitive load was caused by a simple 3D model on which depth cues in the form of simplified shadows were presented. The cognitive load ratings of the most detailed rendering are significantly higher than those of the visualization featuring depth cues. This result could indicate that there may be an optimal level of realism that spares learners the effort to understand an abstract diagram on the one side and that does not overwhelm them with too much detail (see Skulmowski 2022a).

Consequently, the results of both studies do not support an approach based on the idea that contour lines are the most favorable mode of presentation for perception and learning. Although it may be true that humans have evolved mechanisms to “unpack” a considerable amount of information from relatively simple schematic drawings (Hertzmann. 2020), this “unpacking” process appears to induce substantial cognitive load. Thus, at least for tasks in which shapes are to be learned, learners appear to benefit from depth cues (through a reduction in cognitive load as shown in Experiment 2) and may profit from (a restrained level of) surface detail (as demonstrated in Experiment 1).

In sum, the results of the experiments further underline the complexity of the factors that can affect whether learners benefit from learning with visualizations. At least for retention-oriented tasks in which concrete shapes are to be memorized, the current studies highlight that a strong simplification may induce cognitive load and thus may not help learners. A higher level of detail resulted in better learning in one study, but raised cognitive load in the second experiment. Judging from these results, it may be the safest choice to use visualizations that offer depth cues and shadows without fine-grained details.

### Limitations and outlook

It needs to be noted that the studies presented in this article are focused on a rather specific type of learning task which is primarily concerned with memorizing specific (parts of) shapes and their names. Although such tasks can be found in various subjects other than in biology and anatomy education, it has been argued that other tasks, such as learning about processes or more abstract knowledge, can have different demands, and thus might not benefit from realism (Skulmowski et al. 2022). As a result, the effects of the studies need to be replicated with other contents and knowledge types. Furthermore, the studies only considered retention performance. Future studies should investigate more complex arrangements in which more than one structure is presented to assess whether the positive effects of realistic visual properties can still be found in situations in which higher demands through multiple objects are being placed on learners. Furthermore, the exact mechanism behind the positive effects of depth cues and details should be further investigated.

### Conclusion

The two studies provide orientation for the design of instructional computer-generated visualizations by demonstrating that realistic details do not require additional assistance in order for learners to benefit from them. Depth cues and details appear to contribute toward the positive effects of realism. Although it may be easier to process schematic visualizations, it may be difficult to extract the necessary information from contour drawings in order to be able to construct a 3D mental model. As a result, it may be advisable to add at least simplified forms of shading—but not necessarily details—to instructional visualizations.

### Supplementary Information

Below is the link to the electronic supplementary material.

Supplementary file1 (PDF 24 KB)

## Data Availability

The data of these studies are available from the author upon request.

## Footnotes

1 Originally, an even more conservative effect size of *η*_p_^2^ = 0.06 was targeted, but this needed to be adjusted due to concerns over the Omicron variant of SARS-CoV-2.
