# Supplementary material for: Are realistic details important for learning with visualizations or can depth cues provide sufficient guidance?
Source: Cogn Process. 2024 Mar 21;25(3):351–61. doi: 10.1007/s10339-024-01183-3 (PMC11269326; doi:10.1007/s10339-024-01183-3)
Supplement: Supplementary file 1 — Supplementary file1 (PDF 24 KB) [file 10339_2024_1183_MOESM1_ESM.pdf]

Original pre-training text (in German)
Die Ohrspeicheldrüse (Parotis) liegt beidseitig unter und vor dem Ohr. Der größte Teil dieser Drüse weist eine flache Form auf, die nach oben einen breiteren Auswuchs aufweist und nach unten hin schmal zusammenläuft. Aus dieser unregelmäßig geformten Struktur wächst der Parotisgang.
Auf der Oberfläche der Ohrspeicheldrüse und durch sie hindurch verlaufen verschiedene Arterien, Venen und Adern. Auf dem größten Teil der Drüse liegt die Temporalarterie, daneben die Oberkieferarterie. Im unteren Bereich entspringen die Halsschlagader und die Gesichtsvene.
Es gibt auf der Ohrspeicheldrüse mehrere Abdrücke für Schädelknochen und andere Strukturen. So weist die Drüse im oberen Bereich den Eindruck für den Gehörgang auf. Im mittleren Bereich liegt der Abdruck für den Griffelfortsatz. Im unteren Bereich gibt es Abdrücke für den Unterkieferknochen und für den Digastricusmuskel.

English translation
The parotid gland is located below and in front of the ear on both sides. The largest part of this gland has a flat shape and features a wider process towards the top while tapering at the bottom. From this irregularly-shaped structure, the parotid duct grows out.
Various arteries, veins, and blood vessels cling to the surface of the parotid gland or pass through it. On the largest part of the gland, the temporal artery can be found, next to the maxillary artery. In the lower area, the carotis and the facial vein originate.
There are several impressions on the parotid gland from bones of the skull and other structures. In the upper part of the gland, an impression for the auditory canal can be found. In the middle area, there is an impression for the styloid process. In the lower area, there are impressions for the mandible and the digastric muscle.
